# Impact of CT calibration curves on radiotherapy planning using diagnostic CT scans and a planning CT supporting DirectDensity reconstruction

**DOI:** 10.1002/acm2.70522

**Published:** 2026-02-24

**Authors:** Fabian Krause, Frank‐André Siebert

**Affiliations:** ^1^ Department of Radiotherapy University Medical Center Schleswig‐Holstein Kiel Germany

**Keywords:** adaptive radiotherapy, CT calibration curve, DirectDensity, end‐to‐end study, sim‐free radiation

## Abstract

**Purpose:**

DirectDensity enables tube voltage‐independent, density‐calibrated computed tomography (CT) images for treatment planning and is therefore increasingly used in radiotherapy facilities. In accelerated emergency workflows, diagnostic CT (dCT) data are often acquired without DirectDensity. It remains unclear whether using a DirectDensity calibration curve in this context leads to major dose deviations. This study therefore investigates the impact of different CT calibration curves on treatment planning for an artificial intelligence‐supported emergency workflow in a phantom‐based end‐to‐end test. To validate the transferability of phantom measurements to clinical scenarios, a clinically implemented emergency radiotherapy workflow using diagnostic CT data is retrospectively evaluated in four previously treated patients representing different electron density scenarios.

**Method:**

On Varian's Ethos 1.1 platform (Palo Alto, CA, USA), the entire treatment chain was simulated in an adaptive workflow using an Alderson‐Rando phantom scanned on three diagnostic CTs. This simulation, which lacks a dedicated calibration curve for the treatment planning software, was conducted as an end‐to‐end test and was compared with the results of a radiotherapy‐calibrated planning CT scan. The phantom featured a purpose‐built insert for a Farmer 30013 chamber from PTW (Freiburg, Germany). The end‐to‐end test was performed using three different calibration curves (DirectDensity, 120 kV, and a custom hybrid of DirectDensity and 120 kV). Gamma analysis was performed for the retrospectively evaluated patient plans (calculated with the three different CT curves) using three different gamma criteria to assess the robustness of dose agreement under clinically realistic conditions.

**Results:**

The point doses at the effective point of measurement of the ionization chamber, determined using the treatment planning software, were compared with the measured dose values. All tested approaches met the requirements of ICRU Report 24 (± 5% of the prescribed dose) and could be used in emergency workflows with dCTs. Despite minor differences (0.1%–0.7% better accuracy), the hybrid curve achieved the best results. Recalculations of four representative patients confirmed the trends observed in phantom measurements. 2D array evaluations showed that dose differences between calibration curves remained within clinically defined acceptance limits, with the hybrid curve consistently performing best.

**Conclusion:**

Using a DirectDensity calibration curve in emergency workflows with image data acquired without DirectDensity leads to clinically acceptable treatment plans. Dose deviations can be reduced by carefully evaluating the entire treatment chain. A self‐generated hybrid CT density curve shows the best results, although deviations remain larger (< 1.5%) than when planning directly on a calibrated planning CT. The combined phantom‐ and patient‐based analysis demonstrates that calibration curve selection can influence dose distributions under clinically realistic conditions, supporting the superior performance of the hybrid curve and highlighting clinical relevance beyond technical feasibility.

## 1 Introduction

DirectDensity (DD) is an image reconstruction approach offering tube voltage independent density calibrated computed tomography (CT) images for treatment planning in radiotherapy.^1^ Unlike post‐processing methods such as the generation of synthetic CT images, DD is applied directly during image reconstruction from the raw CT projection data. Particularly for dose calculation within the radiotherapy treatment planning systems (TPS), this presents an advantage, as it enables the use of a single calibration curve regardless of tube voltage.^2^ Unlike the conventional CT calibration curve, which converts Hounsfield units (HU) to electron density, DD reconstructs the CT projection data directly into CT numbers that represent the materials’ electron density, which is essential for accurate dose calculation in radiotherapy. Because of this advantage, CT scanners utilizing this method are increasingly used in radiation therapy.

The combination of Artificial Intelligence (AI)‐supported radiotherapy systems^3^, ^4^, accelerated workflows^5^, ^6^, ^7^, and established palliative treatment protocols (e.g., 8 Gy in a single fraction radiotherapy treatment^8^, ^9^) reduces treatment delays and duration, enabling same‐day therapy and gaining adoption. Although online adaptation is helpful for such workflows, it is not a prerequisite and these workflows can also be applied on conventional C‐arm linear accelerators (LINACs).^10^ These treatment approaches typically utilize diagnostic CT (dCT) images for radiation treatment planning and are used in particular for emergency radiation treatments, as they avoid the time‐consuming step of acquiring a separate planning CT (pCT), which is usually required in standard radiotherapy.

As the dCTs are typically not used for radiotherapy treatment planning, the dCT calibration curves are generally not generated in the TPS. Instead, the calibration curve from a previously calibrated pCT scanner is used. Most conventional dCT scanners do not support DD reconstruction. The question therefore arises whether the use of a DD calibration curve in emergency irradiation leads to dose calculation differences beyond those observed with a standard 120 kV calibration curve. While dose deviations within established quality assurance criteria (e.g., ± 5% of the prescribed dose) are generally considered clinically acceptable, such agreement does not necessarily imply dosimetric equivalence. Systematic trends in CT calibration curve selection may enhance clinical robustness in adaptive emergency workflows, where robustness is critical. Feliciani et al.^11^ characterized the possible error in the selection of the correct calibration curve within the same reconstruction algorithm. To the best of our knowledge, no peer‐reviewed study has been published comparing DD calibration curves with 120 kV calibration curves in an end‐to‐end test for an emergency radiation workflow, which addresses the extent of possible uncertainties between the two methods. However, this question is interesting for several reasons.

If using a CT calibration curve derived from DD reconstruction in combination with a dCT acquired without DD reconstruction results in only minor deviations in dose calculation then standardizing to a single calibration curve could help prevent errors due to incorrect curve selection in emergency irradiation scenarios. In the presence of clinically relevant deviations, not using a second calibration curve may compromise the robustness of the dose calculation. The results may also support the development of a dedicated calibration curve for emergency irradiation, thereby helping to reduce overall uncertainty, where a second calibration curve is helpful.

This study investigates the impact of the CT calibration curve selection on treatment planning when using uncalibrated dCT data from a CT scanner, not supporting DD reconstruction. The entire treatment planning chain is checked with an Alderson phantom in an end‐to‐end test with dosimetric measurements for an AI supported adaptive workflow. Different diagnostic CTs are used and the results are compared with the results of a pCT calibrated for radiotherapy. Calibration curves based on both DD reconstruction and conventional CT reconstruction are compared, and a novel hybrid calibration curve is proposed to enhance the current approach.

To evaluate the influence of CT calibration curves under realistic clinical conditions, representative treatment plans from a clinically implemented emergency workflow are retrospectively recalculated using the different calibration curves previously described. Unlike a purely technical workflow description, this study assesses their dose‐related effects using real patient data.

## 2 Methods

### 2.1 Experimental setup

The complete radiotherapy workflow, including image acquisition and simulation as well as patient setup and dose delivery, must meet the ICRU Report 24^12^ accuracy requirement of ± 5% of the prescribed dose. To verify compliance in an AI‐supported adaptive workflow, the whole treatment chain was checked utilizing a modified male Alderson‐Rando phantom,^13^ consistent with a previously established experimental setup,^14^ by which the feasibility of a dCT‐based workflow, without considering the potential differences resulting from the selection of different calibration methods for dose calculation, was evaluated. In this setup, further CT calibration procedures were performed, applied during dose calculation and examined for their influence. Although ICRU Report 24 was published as early as 1976, many modern audit and end‐to‐end test publications continue to reference its recommended 5% target for the accuracy of absorbed dose.^15^, ^16^, ^17^ We also find this recommendation useful in defining the tolerance range for an emergency irradiation, not least because this 5% criterion is also in accordance with the AAPM MPPG 5.b,^18^ which allows dose deviations of up to ± 5% within the high dose region for the verification of complex configurations during TPS commissioning. Emergency workflows correspond to rather complex, non‐ideal conditions.

**FIGURE 1:**
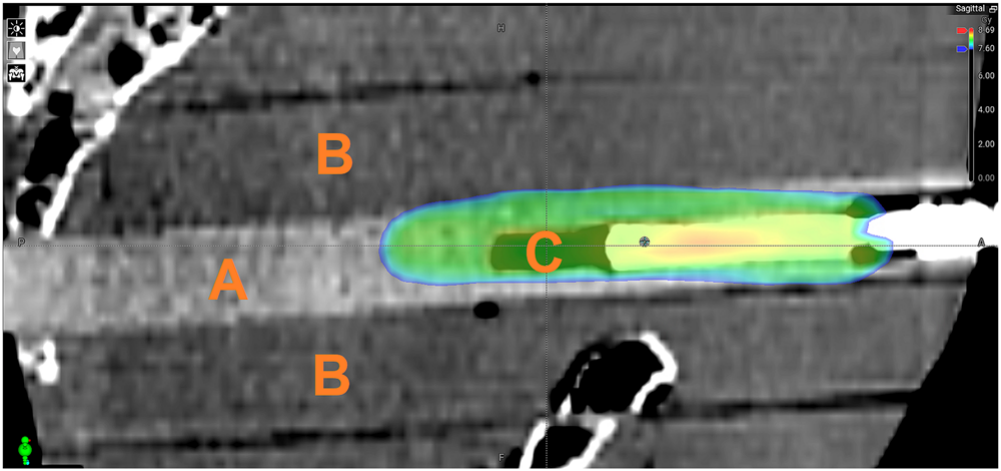
Purpose‐built PMMA Farmer chamber holder (A) in sagittal sCT view within in the Alderson phantom (A) with a homogeneous dose distribution (V95% displayed) over the ionization volume (C) of the measurement chamber. PMMA: polymethyl methacrylate; sCT: synthetic computed tomography; V95%: volume receiving ≥95% of the prescribed dose.

Dose calculation was based on the data of a pCT scanner with its associated calibration curves, which were also applied to three different dCT scanners. For dose calculation, both pCT and dCT image data were used and compared with each other. Dose verification was performed using a Farmer ionization chamber type 30013 (PTW, Freiburg, Germany) placed in a purpose‐built polymethyl methacrylate (PMMA) holder (Figure 1). Although the Farmer chamber has a relatively large sensitive volume compared to smaller detectors such as PinPoint or Semiflex chambers, it was chosen in this setup due to its low angular dependence.^19^

While the Alderson phantom lacks physiologically driven motion (like organ shifts) and cannot fully replace real human tissue, it serves as a comprehensive surrogate for dose measurements approximately corresponding to those in real patients. This study focuses primarily on the effects on physical dose distribution and less on the effects related to physiological processes. The phantom's rigid, tissue‐equivalent materials enable reproducible measurements, with the absence of physiological processes even being an advantage, as dose differences can be clearly attributed to the different imaging systems used.

### 2.2 Treatment chain and end‐to‐end workflow

The entire treatment planning chain comprises a SOMATOM go.Sim dual‐energy CT (Siemens Healthineers, Erlangen, Germany), the Ethos Treatment Management System v1.1 using the algorithm Ethos Acuros XB v1.1.1001 (Varian, Palo Alto, USA) for dose calculation, and an Ethos Linear Accelerator (Varian) with a 6 Megavolt flattening filter free photon beam.

**FIGURE 2:**
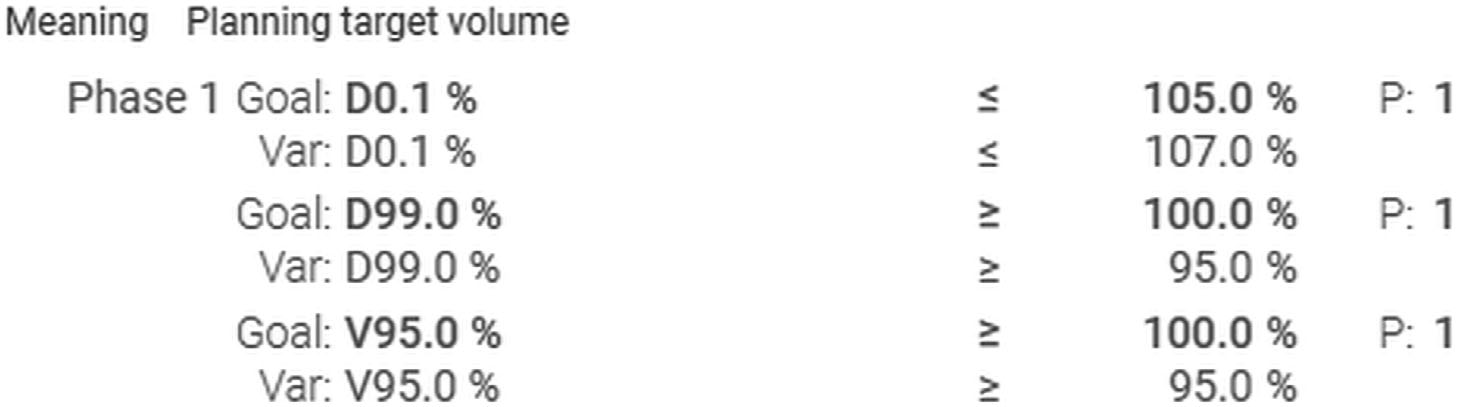
Optimization criteria for dose calculation defining the objectives, constraints and weighting factors. D0.1%: 0.1% of the volume received this dose or more; D99%: Dose received by 99% of the volume; P: Weighting factor. V95%: Volume receiving ≥95% of the prescribed dose.

A CT scan of the Alderson phantom was conducted using the SOMATOM go.Sim with DD enabled, and the data were transferred into the TPS. Since the density values of the sCTs generated as a result of the adaptive workflow depend on the initial planning imaging, it is desirable to generate images free of artifacts for the treatment planning process. To reduce metal induced artifacts, the Farmer ionization chamber was removed during scanning. A radiation treatment plan (8 Gy per fraction) with the associated DD calibration curve was then created using Ethos Treatment Management with the constraints given in Figure 2. The treatment planning technique resembles standard clinical practice. For the equivalence across all CT datasets, a nine‐field sliding window intensity‐modulated radiotherapy (IMRT) plan was used for all calculations to ensure better comparability. This approach was chosen because IMRT enables faster online adaptive planning than volumetric modulated arc therapy, providing a good balance between plan quality, time efficient planning, and patient comfort (particularly important in emergency radiotherapy with limited on‐couch time). In contrast to conventional emergency treatments, which are often delivered using three‐dimensional (3D) conformal radiotherapy techniques (CRT) with a strong anteroposterior‐posteroanterior beam arrangement, organ at risk sparing IMRT plans represent the current standard in online adaptive workflows.^5^ The Varian Ethos system is based on a strict single‐console workflow, making 3D‐CRT planning practically impossible without ARIA interaction. Introducing 3D‐CRT would require additional manual steps and external ARIA workflows, increasing treatment time and therefore contradicting the intended emergency treatment workflow.

A 50 cm^3^ planning target volume (PTV) was defined to fully cover the Farmer chamber's sensitive volume of 0.6 cm^3^ and to ensure a uniform dose distribution within the measurement volume. The dose distribution was similar to a typical palliative patient case with a homogeneous prescription within the PTV. Since the Farmer chamber's sensitive volume contains air, no density overrides were applied during treatment planning, ensuring measured doses accurately matched TPS calculations without manual heterogeneity corrections. Afterwards, the phantom, with the Farmer chamber inserted, was positioned on the linear accelerator, and an adaptive workflow was initiated. Individual adaptive plans were then calculated and delivered for each fraction, while the dose was being measured. The procedure was repeated ten times to evaluate statistical variability. The planned dose at the reference point within the Farmer chamber in the adaptive plan's sCT was compared to the measurements. The point dose at the chamber's reference location (1.30 cm from the tip along the chamber axis per PTW datasheet) was used, because it was located at the center of both the IMRT plan fields and the ion chamber. This approach simplified the adaptive workflow, as the dose is nearly homogeneous within the chamber's sensitive volume. A comparison between the point dose method and the integration over the entire sensitive volume of the chamber was carried out based on ten measurements and showed minor deviations of 0.4% in average. At this point, it remains unclear whether this slight deviation stems from the methodological approach or from the resolution of the computational grid.

**FIGURE 3:**
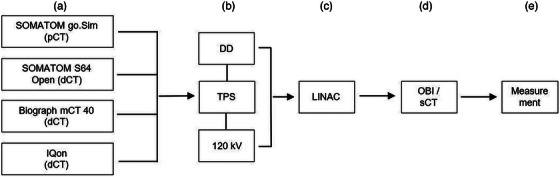
Illustrated workflow: a) Acquisition of phantom CT data and import of CT data into the TPS for subsequent plan calculation; b) Simulation in the TPS (plan calculation in the TPS was performed using both the DD calibration curve and the conventional 120 kV calibration curve, with all subsequent workflow steps carried out for plans generated with both calibration curves); c) Data transmission of calculated phantom treatment plans to the LINAC; d) Use of onboard imaging (OBI) for acquiring CBCT used for creation of phantom sCT; e) Dose measurement in phantom setup with ion chamber and evaluation against dose calculated on phantom sCT. CT: Computed tomography; dCT: Diagnostic computed tomography; DD: DirectDensity; kV: Kilo voltage; LINAC: Linear accelerator; OBI: Onboard imaging; pCT: Planning computed tomography; sCT: Synthetic computed tomography; TPS: Treatment planning system.

The workflow was repeated multiple times using the following diagnostic CT scanners instead of the SOMATOM go.Sim to generate dCTs for plan calculation and creation of an adaptive sCT: SOMATOM Sensation 64 Open (single‐energy CT by Siemens), Biograph mCT 40 (PET/CT scanner by Siemens), and Philips IQon (Spectral CT by Philips, The Netherlands, Amsterdam). The standard abdomen/pelvis protocol of each scanner was used, utilizing a consistent tube voltage of 120 kV and slice thicknesses ranging from 0.1 to 0.3 cm. This setup was used to evaluate the entire treatment planning chain of an accelerated workflow based on diagnostic images using a DD‐based CT calibration curve. In order to enable comparison with a conventional 120 kV CT calibration curve and to examine for differences, the previous steps were repeated for the diagnostic CT scanners, with the difference that a 120 kV calibration curve for the SOMATOM go.Sim was used for the initial plan creation, and the results were compared with the previous ones (Figure 3). A subset of the data (generated using the DD reconstruction) has been previously presented in a different context.^14^

An uncertainty analysis for our evaluation, regarding dose calculation in the TPS and dose measurement uncertainties (including those for the Farmer chamber, detector positioning, and dose calibration), is presented in the results section. The combined standard uncertainty was calculated assuming uncorrelated uncertainty contributions using the root‐sum‐of‐squares method.

### 2.3 CT calibration curves

CT number (HU) depend on the specific CT scanner and tube voltage used. To account for these influences and associated uncertainties, the treatment planning system uses a device‐specific CT calibration curve, created by scanning a phantom with known density inserts, that relates HU values ​​to electron/physical densities to enable accurate dose calculation. To compare the influence of the tube voltage–independent reconstruction approach DD when planning on unspecified diagnostic CT images acquired with scanners not supporting DD reconstruction, a calibration curve for the department‐specific planning CT SOMATOM go.Sim (by Siemens) was created using raw data reconstructed with DD (indicated as Sd40 by the SOMATOM) and with the standard convolution kernel (indicated as Qr40). A Gammex electron density CT phantom, model 465 (Gammex Inc., Middleton, WI, USA), was used to create the calibration curves.

**FIGURE 4:**
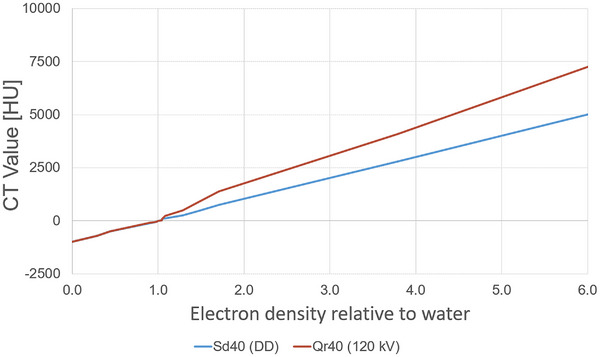
Comparison of the different CT calibration curves. CT: Computed tomography; DD: DirectDensity; HU: Houndsfield unit; kV: Kilo voltage.

The DD calibration data were acquired according to the DD cookbook^20^ by Siemens Healthineers. During the calibration procedure, repeated scans at tube voltages of 70, 80, 100, 120 and 140 kV were systematically acquired, and the average DD value was obtained for each insert by averaging the corresponding CT values. The Qr40 calibration curve is based solely on the 120 kV scan, which is standard practice in external beam radiation therapy using photons. Figure 4 shows a comparison of the two calibration curves.

### 2.4 CT calibration curve evaluation in emergency radiotherapy patients

An emergency radiotherapy workflow integrating purely diagnostic CT data into online adaptive treatment based on the evaluated workflow was clinically implemented in our department two years ago. The initial patient cohort was treated using this approach as part of routine emergency oncologic care. To independently validate the influence of CT calibration curve selection beyond the phantom‐based evaluation, a retrospective analysis of real patient treatment plans was performed including four previously treated patients. The patients represented a small but diverse cohort covering clinically relevant electron density scenarios, rather than aiming to perform statistical inference: pelvic bone, abdominal soft tissue, thoracic soft tissue and thoracic bone. All patients were treated using the clinically implemented accelerated emergency workflow. The diagnostic CT scanners used for the creation of these patient plans were neither part of the phantom measurements nor the development of the subsequently introduced hybrid calibration curve. This deliberate separation ensured an independent validation of the calibration approaches under realistic clinical conditions. All planning parameters, including beam geometry, monitor units, dose calculation algorithm and raster resolution remained unchanged. The CT calibration curve was the only variable used in the recalculations. Dose application was verified using a 2D detector array (OCTAVIUS 1500 by PTW Freiburg GmbH, Germany) in a phantom setup and dose differences between the calibration approaches were evaluated using two‐dimensional (2D) gamma analysis. Planar 2D gamma analysis enabled an independent and reproducible assessment of spatial dose differences without introducing additional uncertainties related to 3D dose reconstruction, including algorithmic interpolation effects. This approach ensured that observed deviations were directly attributed to differences in the CT calibration curves and also reflects the routine clinical, patient‐specific quality assurance of IMRT plans. Gamma analysis was performed using PTW VeriSoft with local dose normalization and a dose threshold of 10%. The measured dose map served as a reference for pairwise comparisons to assess spatial dose agreement in the different scenarios. Three gamma criteria were applied: 3 mm/3% as a historical benchmark, 2 mm/3% reflecting AAPM Report TG‐218 and enabling the assessment of more subtle spatial differences potentially arising from variations in CT calibration curves and 2 mm/2% to explore the limitations of the chosen approach. According to AAPM Report TG‐218, the tolerance limit for gamma analysis at 2 mm/3% is ≥95% and represents a stringent criterion for clinical quality assurance, whereas the intervention limit is ≥90% and indicates the minimum acceptable level at which corrective action is required. While TG‐218 recommends global normalization for routine IMRT QA, gamma analysis was performed using local dose normalization to increase sensitivity to relative dose differences between CT calibration approaches and to provide a stricter, more conservative assessment, ensuring that plans exceeding the action limit of 90% are safely within clinical acceptability. Given the emergency workflow and the use of diagnostic CT scans, a gamma pass rate of ≥ 90% at 2 mm/3% was therefore considered acceptable.^21^ For this study, treatment plans of previously treated patients were retrospectively analyzed, with the local ethics committee having approved the retrospective data analysis.

## 3 Results

### 3.1 Results of end‐to‐end test

For DD pCTs, the DD calibration curve is considered the gold standard, as DD‐supported reconstruction kernels directly convert HU into relative electron densities, ensuring accurate patient‐specific dose calculation. Using a conventional 120 kV calibration curve would require a new CT reconstruction with an alternative kernel from the raw data, which is not typically performed in standard DD workflows. Therefore, only values derived from DD reconstruction are reported below for the pCT.

**FIGURE 5:**
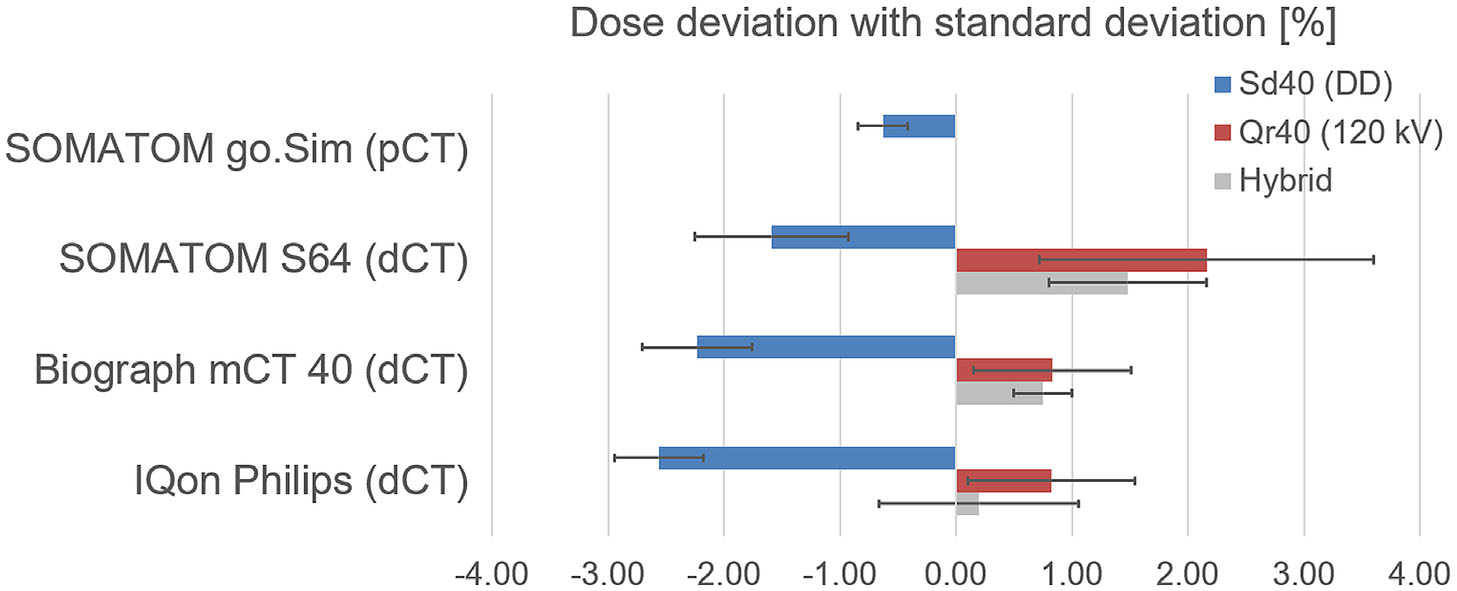
Dose deviation (mean ± standard deviation) between the TPS‐calculated point dose and the corresponding Farmer ionization chamber measurement for the selected CT scanners (including a hybrid approach that was evaluated based on the findings described below). All deviations are reported as a percentage relative to the measured point dose, representing the absolute dose calculation accuracy. Negative values indicate an underestimation and positive values an overestimation of the measured dose. dCT: Diagnostic computed tomography; DD: Direct Density; kV: Kilo voltage; pCT: planning computed tomography.

The use of diagnostic images influences dose calculation in the TPS. Treatment plans based on calibrated pCT‐data show minor deviations from measurements than those using dCT‐data (Figure 5). Despite a slight dose underestimation, dose calculations with the Acuros XB algorithm on pCT‐data agree well with measurements (average deviation −0.6 ± 0.2%). The underestimation increases when calculating plans on dCT‐data using the Sd40 (DD) calibration curve but remains within the defined accuracy requirement of ± 5% of the prescribed dose for all three uncalibrated diagnostic devices. In contrast, using the Qr40 calibration curve obtained at 120 kV results in an overestimation of the dose compared to the measured reference values, which still fall within the predefined ± 5% tolerance limit.

No clear advantage of either calibration curve is observed across all dCTs considered. However, considering all dCTs, the Qr40 curve shows slight overall advantages (in two out of three dCTs) when compared to the ion chamber measurements. For both the Biograph mCT 40 and the IQon the absolute deviation from the measured dose values is lower compared to the DD calibration curve.

The dose calculation in the TPS is based on converting HU values into density or electron density. Higher HU values typically indicate denser materials, which cause greater attenuation of the radiation beam. To compensate for this, the TPS plans more monitor units (MU) to ensure adequate irradiation of the target volume. In the present case, the situation is as follows: for a given HU value in the CT, the flatter curve Sd40 (DD) assigns a higher density. When using the flatter curve, the TPS interprets even small HU increases as larger changes in density. As a result, the tissue is assumed to be denser. In contrast, the steeper Qr40 (120 kV) curve assigns a lower density for the same HU value, interpreting larger HU increases as smaller changes in density compared to the flatter curve.

Considering the CT calibration curves from Figure 4 in this context, the results presented in Figure 5 can be explained. While the calibration curve for the Sd40 (DD) reconstruction shows lower HU values for electron densities greater than 1 relative to water, the calibration curve for the Qr40 (120 kV) reconstruction shows higher HU values in the same range. The same applies to the curve for equivalent physical density. For the Qr40 (120 kV) curve the same HU value corresponds to a lower estimated density. The same effect occurs when the HU increases. As a result, the TPS reduces the MU to account for the underestimated absorption, leading to a lower measured dose in the target volume and an overprediction of the dose by the TPS. Conversely, the Sd40 (DD) curve exhibits the opposite behavior.

As one calibration curve results in a systematic dose underestimation and the other in a systematic overestimation, the corresponding dose estimates were interpreted as bounding the expected dose range. For this reason, a third hybrid calibration curve was generated by arithmetic averaging of the two previous curves (Table 1). The hybrid calibration curve can be seen as a pragmatic approach to mitigate systematic bias rather than as a claim of exact dose accuracy. The hybrid HU values represent the arithmetic mean of the HU values obtained from the DD and 120 kV calibration curves, calculated as the sum of both values divided by two (rounded).

**TABLE 1:** Phantom inserts with corresponding HU values used for ‘hybrid’ curve. High‐density region subsequently extrapolated according to the Siemens cookbook.

*Material*	*Physical density [g/cc]*	*RED (relative to water)*	*HU value DD*	*HU value 120 kV*	*HU value Hybrid*
True air	0.001	0.000	−981	−983	−982
LN‐300 Lung	0.300	0.292	−705	−708	−706
LN‐450 Lung	0.450	0.438	−505	−506	−505
AP6 adipose	0.920	0.895	−114	−105	−109
BR‐SR1 breast	0.990	0.980	−54	−48	−51
True water	1.000	1.000	1	2	1
BRN‐SR2 brain	1.045	1.039	10	22	16
LV‐1 liver	1.080	1.050	82	86	84
IB Inner bone	1.120	1.081	118	233	175
CB2‐30% CaCO_3_	1.340	1.285	253	493	373
CB2‐50% CaCO_3_	1.560	1.473	475	897	686
SB3 Cortical Bone	1.84	1.707	749	1381	1065

Abbreviations: DD: DirectDensity; HU: Houndsfield unit; kV: Kilo voltage; RED: Relative electron density.

To evaluate whether this method offers any advantage, the end‐to‐end test is repeated with the third calibration curve using the same procedure as before. The results are shown in Figure 5.

**FIGURE 6:**
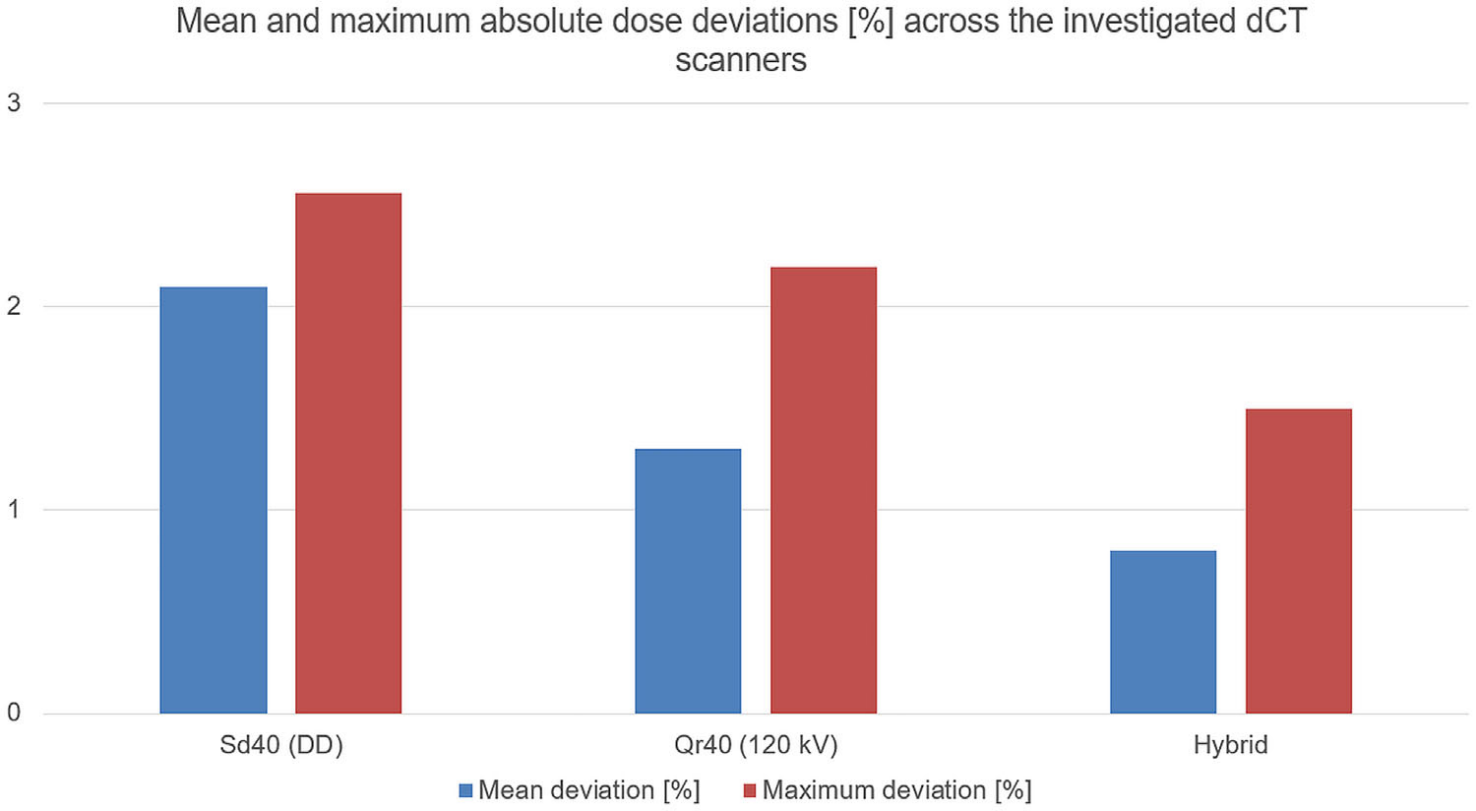
Comparison of mean and maximum absolute dose deviations in percent for the evaluated calibration curves (Sd40, Qr40, and Hybrid) across the considered dCT scanners. Mean deviations indicate systematic trends, while maximum deviations are given ​​to highlight worst‐case behavior. dCT: Diagnostic computed tomography; DD: DirectDensity; kV: Kilo voltage.

The results shown in Figure 5 indicate that the hybrid curve reduces dose overestimation compared to the use of the Qr40 curve across all three CT scanners examined. The deviation from the values measured with the ionization chamber is also smaller for all three dCTs compared to all previously used curves. However, the differences are sometimes small (for example, +0.8% for the Biograph mCT 40 when using the Qr40 curve, and +0.7% when using the hybrid curve). The hybrid curve shows the best results across all measurements and all three dCTs considered and remains within the defined accuracy requirement of ± 5%. When the measured dose values are used as the reference for comparison, the maximum overall deviation decreases from over 2% (Sd40 and Qr40) to 1.5% for the hybrid curve (Figure 6).

When considering the mean deviations as the average of all three dCTs examined, the previously observed systematic trend toward the differences between the different calibration curves becomes even more apparent (Figure 6). Although all three calibration curves meet the requirement of ± 5%, systematic trends can be identified, which indicate the superiority of the hybrid approach.

### 3.2 Patient data

To assess the influence of the choice of CT calibration curve, recalculations are performed for four representative patients who had undergone the clinically implemented accelerated emergency workflow. For each patient, the originally clinically approved treatment plan is recalculated using three different CT calibration curves (DirectDensity, 120 kV and hybrid curve). The 2D array measurements shown in Table 2 confirm the trends observed in the phantom point dose analysis. The differences between the calibration curves all remain within the clinically defined acceptance limits described in Section 3.4 and they confirm the previously observed superior performance of the hybrid curve.

**TABLE 2:** Retrospective two‐dimensional verification of patient treatment plans using multiple CT calibration curves with different gamma analysis criteria.

*Patient ID*	*Anatomical site*	*Calibration curve*	*Gamma (3mm/3%)*	*Gamma (3mm/2%)*	*Gamma (2mm/2%)*
1	thoracic (low density/lung tissue)	DirectDensity	94.8	90.7	80.2
		120 kV	97.6	95.6	89.5
		Hybrid	98.0	96.4	89.5
2	abdominal soft tissue (moderate density/spleen)	DirectDensity	93.9	91.3	77.3
		120 kV	97.4	96.5	88.6
		Hybrid	98.3	97.4	90,4
3	pelvic bone (high density/cortical tissue)	DirectDensity	97.3	96.3	79,3
		120 kV	98.4	98.4	87,2
		Hybrid	98.9	98.9	92,0
4	thoracic bone (high density/low density)	DirectDensity	96,4	94,8	71,2
		120 kV	96,8	96,4	74,0
		Hybrid	97,6	96,8	82,4

Abbreviations: ID: Identification; kV: Kilo voltage.

Using the historical criterion of 3 mm/3%, all plans achieve a pass rate of over 93%, confirming compliance with routine clinical quality assurance. Using the stricter criterion of 3 mm/2%, the 120 kV and hybrid calibration curves consistently achieve pass rates of ≥95%, thus meeting the tolerance limit defined by AAPM TG‐218, while the DD curve shows slightly lower performance in some cases but still achieves passing rates over 90%, which is defined as sufficient based on the criteria set in Section 3.4. The strictest criterion of 2 mm/2% highlights the limitations of the method, with none of the evaluated calibration curves achieving results over 90% for all evaluated cases. Gamma analysis shows improved agreement for the hybrid calibration curve, particularly in areas of higher electron density, as is observed in the 2 mm/2% gamma analysis for the thoracic and pelvic bone case.

**FIGURE 7:**
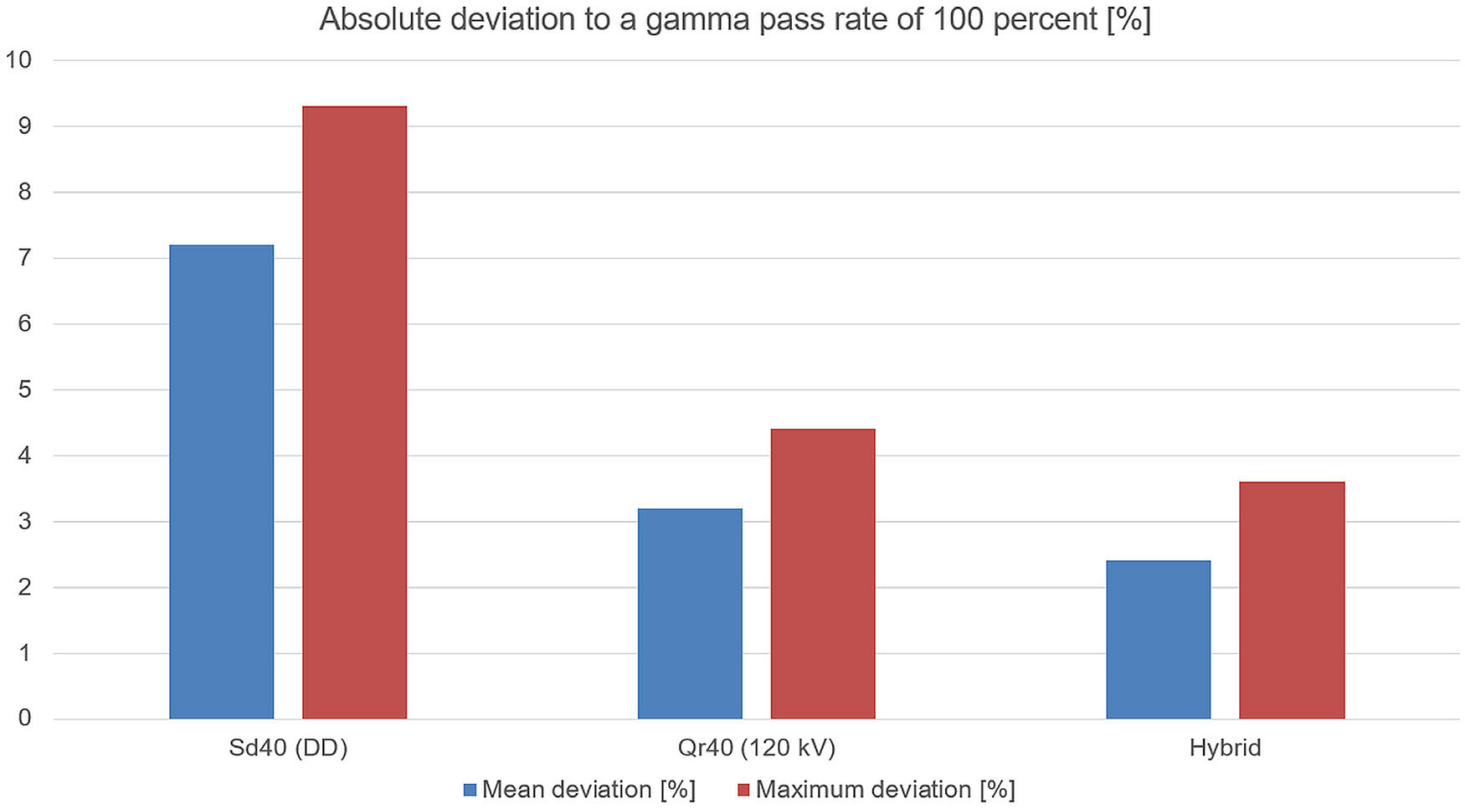
Mean and maximum absolute deviation to a gamma pass rate of 100% (2 mm/3% criterion) for the three calibration curves (DD, 120 kV and Hybrid). Deviations are shown relative to an ideal gamma pass rate of 100%. Lower values indicate improved agreement between calculated and measured dose distributions. DD: DirectDensity; kV: Kilo voltage.

When comparing the mean absolute point dose deviations averaged over the three investigated dCT scanners (Figure 6) with the mean absolute deviation from a gamma pass rate of 100% (2 mm/3%) averaged over the four evaluated patient plans (Figure 7), consistent trends across the different calibration curves are observed. The same trend is observed for the maximum deviations. This agreement demonstrates that the effect of calibration curve choice propagates consistently from localized point dose deviations to spatially resolved dose distribution accuracy.

### 3.3 Uncertainty analysis

To assess the overall uncertainty of the end‐to‐end test, each step of the workflow is systematically evaluated and quantified, providing detailed insight into reliability and accuracy (Table 3).

**TABLE 3:** Individual uncertainties and combined standard uncertainty (*k* = 1), calculated assuming uncorrelated contributions.

*Procedure*	*Origin of uncertainty*	*Type*	*Uncertainty*
Dose calculation	Determination of dose in the TPS	B	0.4%
Dosimetry	Uncertainty of Farmer chamber	B	1.6%
	Position in chamber	A	0.5%
	Dose calibration	B	2.0% (1.0%)
*Combined standard uncertainty 2.6% (2.0%)*			

Abbrevation: TPS: Treatment planning system.

As described in Section 3.2, a comparison between the point dose method and the integration over the entire sensitive volume of the chamber was carried out based on ten measurements. This showed small deviations of 0.4%. Therefore, a systematic error of 0.4% is estimated as the uncertainty in the determination of the dose in the TPS. Based on the findings of Castro et al.^22^, the absorbed dose to water measured with a Farmer chamber carries an uncertainty of approximately 1.6%. An error of 0.02 cm was estimated for the uncertainty in finding the effective measurement point inside the ionization chamber. This leads to a dose uncertainty of 0.5%. By conducting periodic in‐house quality assurance, the stability and accuracy of the calibration process are ensured, allowing for the detection and correction of any potential deviations over time and thereby maintaining the reliability of dose delivery within clinically acceptable limits. Although the actual variability in measurements using the Farmer chamber on the Ethos platform was about 1%, a department‐internal adjustment threshold of up to 2% is applied. Accordingly, this value is adopted as the uncertainty in dose calibration to ensure consistency with the department's workflow. When a reduced calibration uncertainty of 1.0% was assumed, the combined standard uncertainty decreased to 2.0%.

## 4 Discussion

The ICRU Report 24 recommends a point dose accuracy of ± 5% for dose delivery to the target volume^12^, a standard that, despite ongoing debate since its 1976 publication, remains widely accepted as balancing clinical needs with technical feasibility.^23^, ^24^ This ± 5% criterion continues to be commonly applied in practice today.^15^, ^16^, ^17^ Furthermore, actual guidelines and reports of internationally respected professional societies refer to it, so it can still be regarded as internationally accepted.^18^, ^25^, ^26^ Modern hypofractionated palliative treatment protocols^8^, ^9^, used in emergency radiotherapy, typically prescribe homogeneous doses within the planning target volume (PTV). Multiple randomized clinical trials have proven the effectiveness of these treatment regimes,^27^ whose dose distribution is well suited to the chosen experimental setup. The comparison between the point dose method and the integrated measurement over the entire sensitive volume of the chamber revealed only minor deviations, which further supports the presence of a homogeneous dose distribution within the PTV. The transferability of the results from point dose measurements to 2D dose maps has been demonstrated before, and it has also been shown that clinically satisfactory 3D gamma results can be expected when point dose deviations are less than 3%.^14^ Therefore, the end‐to‐end test using the point dose method seems to be a suitable and effective technique, providing accurate dose measurements and ensuring treatment quality within the PTV, as it offers an independent verification to prevent dosimetric errors.

DD allows a more energy‐independent calibration.^28^ Even though the dose deviations are clinically acceptable, they increase when using DD. The observed deviation of 0.6% for the reference planning CT is in good agreement with previous findings reported by Flatten et al.^2^ This supports the confidence in the validity of the chosen measurement method. Looking at the results, it is evident that all of the approaches presented here meet the requirements of ICRU Report 24 and could potentially be used in cases of emergency radiotherapy when using dCT‐data. Even though the margins are small (0.1%–0.7% better accuracy referring to the measured dose) across all three dCT scanners examined, the hybrid curve shows the best performance. When using the hybrid curve, the maximum overall deviation relative to the measured dose values decreases from over 2% to 1.5%. The slight small differences between the different calibration curves can be explained by taking a closer look at the physical conditions. The divergence of the curves can be observed at electron densities greater than 1 relative to water (see Figure 5) and is well documented in the literature.^29^, ^30^, ^31^ Like a real patient the Alderson‐Phantom largely consists of tissue‐equivalent material representing a density near one. To realistically simulate radiation absorption in the human skeleton, it also contains bone‐like structures with a density higher than one. The phantom's irradiated pelvic region contains several of these bony structures, with which the rays interact. However, interactions in the tissue‐equivalent material are largely considered, in agreement with clinical reality. Therefore, the influence of the various calibration curves is noticeable, but smaller than one might initially expect from observing the divergent curves.

In addition to dose measurements at phantom points, patient‐based 2D array measurements were performed to evaluate the influence of CT calibration curves under realistic clinical conditions. The evaluation of the retrospectively analyzed patient data confirmed the trends observed in the phantom and thus supports the transferability of the results to clinical scenarios. The agreement between point dose measurements and 2D gamma‐based plan evaluation demonstrates that the observed effects are not measurement‐specific artifacts but arise from systematic density assignment differences. The identified differences in gamma passing rates indicate that the choice of calibration curve can influence dose distributions, particularly in regions of higher electron density. These results support, though not proving, the mechanistic interpretation derived from the phantom study and demonstrate applicability beyond a purely technical workflow evaluation. Despite the small patient cohort, the selection encompassed a range of electron density scenarios relevant to emergency radiotherapy workflows, supporting meaningful conclusions regarding the hybrid calibration curve. Although initially evaluated using phantom measurements, the hybrid curve was independently validated on diagnostic CT scans of patients not included in the phantom cohort. The consistent dosimetric agreement across different anatomical regions underscores the robustness of the hybrid approach and suggests its applicability beyond a single phantom geometry. Based on these findings, the hybrid calibration curve was introduced into clinical use for our sCT‐based workflow, accompanied by enhanced patient‐specific quality assurance.

Even if the recently released version 2.0 of Varian's Ethos platform eliminates the need for an sCT,^32^ the use of sCTs remains available and clinically relevant, since several other adaptive approaches still rely on them.^33^ Even on the Ethos 2.0 platform, the sCT‐based workflow can still be chosen and could be advantageous in certain cases. For example, if reduced CBCT image quality is expected in severely obese patients, the sCT workflow may be advantageous due to its less error‐prone dose calculation.^34^ Especially in patients who are candidates for palliative emergency radiation therapy, CBCT imaging is more likely to show metal artifacts due to previously implanted metallic devices,^35^ which could be an indication to favor the use of an sCT workflow. In these situations, the sCT workflow provides a more robust and error‐resistant alternative, ensuring reliable dose calculation and plan adaptation. Our department has also implemented the Ethos 2.0 platform with the HyperSight imaging detector and continues to use the sCT workflow for these cases, even on the updated Ethos platform. To the best of our knowledge, no peer‑reviewed study has examined the transferability of results between individual manufacturers, but it can be assumed that the findings presented here remain valid as long as dose calculation is based on the pCT calibration curves and the density values are derived from the initial imaging. Furthermore, the question addressed here is also relevant for clinics that have a pCT with a DD module but perform their emergency irradiation on conventional C‑arm accelerators.^10^ In this case too, the generation of the density values used for calculation is based on the initial imaging, so the conclusions regarding dose differences due to calibration curves remain valid, although additional workflow‐specific adjustments may be necessary to account for geometric deformation. Particularly, treatments with conventional C‐arm LINACs may require larger planning margins to compensate for these geometric uncertainties. Furthermore, recent work has demonstrated that online adaptive radiotherapy workflows can be implemented on standard C‑arm LINACs, illustrating that adaptive protocols are not restricted to specific platforms. Such approaches on C‑arm LINACs also typically rely on sCT generation for dose calculation, similar to our sCT‐based workflow. This supports the applicability of our findings beyond the Ethos platform, as the underlying principle remains valid across different platforms.^36^

With the establishment of online adaptive radiation techniques, there is a growing interest in dCT‐based workflows, with the goal of applying them not only in emergency radiation treatments.^37^, ^38^ Consequently, the question of choosing the right workflow and especially the influence of the selected calibration methods for online adaptive simulation‐free irradiation will become increasingly important in the future. The same applies to defining the correct limits for verifying the methods and the allowable deviations.

## 5 Conclusion

Based on the question posed at the beginning regarding dosimetric equivalence in dose calculation, it can be concluded that the use of DD calibration curves for treatment planning with imaging data acquired without a DD module does not lead to clinically unacceptable plans in the cases considered here. Although systematic trends can be observed for the different approaches, no specific curve seems to be strictly required to achieve clinically acceptable results. The decision ultimately depends on clinical priorities, as reflected in the results presented here. However, the results show that the self‐generated hybrid calibration curve consistently delivers the highest gamma pass rates and the most robust dosimetric agreement across all anatomical regions and gamma criteria, followed by the 120 kV curve. The question to consider is whether potential sources of error should be avoided by using alternative calibration curves, or whether dose calculation should be based on a calibration curve that provides more robust agreement in the evaluation of dose differences, even when all approaches remain within clinical acceptance limits. As robustness is a crucial criterion in dCT‐based planning, it was therefore given priority in our evaluation. The fact that the results for the self‐generated hybrid curve provide the best results overall shows that dose deviations can potentially be reduced by examining the entire treatment chain carefully, although the resulting deviations are still on average expected to be larger than those achieved when planning directly on a pCT.

## AUTHOR CONTRIBUTIONS

**Fabian Krause**: Conceptualization; formal analysis; investigation; methodology; project administration; resources; validation; visualization; writing—original draft. **Frank‐André Siebert**: Resources; supervision; writing—review and editing.

## CONFLICT OF INTEREST STATEMENT

The authors declare that they have no conflicts of interest.

## ETHICS APPROVAL

The retrospective data analysis was approved by the local institutional ethics committee (Approval No. D 552/24).

## Data Availability

The data that support the findings of this study are available from the corresponding author on reasonable request.
