# Correction to: “Impact of CT calibration curves on radiotherapy planning using diagnostic CT scans and a planning CT supporting DirectDensity reconstruction”

**DOI:** 10.1002/acm2.70572

**Published:** 2026-04-30

1

Krause F, Siebert F. Impact of CT calibration curves on radiotherapy planning using diagnostic CT scans and a planning CT supporting DirectDensity reconstruction. Appl Clin Med Phys. 2026;e70522. https://doi.org/10.1002/acm2.70522

Using the historical criterion of 3 mm/3%, all plans achieve a pass rate of over 93%, confirming compliance with routine clinical quality assurance. Using the stricter criterion of 2 mm/3%, the 120 kV and hybrid calibration curves consistently achieve pass rates of ≥95%, thus meeting the tolerance limit defined by AAPM TG‐218, while the DD curve shows slightly lower performance in some cases but still achieves passing rates over 90%, which is defined as sufficient based on the criteria set in Section 3.4. The strictest criterion of 2 mm/2% highlights the limitations of the method, with none of the evaluated calibration curves achieving results over 90% for all evaluated cases. Gamma analysis shows improved agreement for the hybrid calibration curve, particularly in areas of higher electron density, as is observed in the 2 mm/2% gamma analysis for the thoracic and pelvic bone case.

## 2 CITATION OF THE ERROR

Table 2 (table header indicating the gamma criterion) and one corresponding sentence in the Results section.

**TABLE 2:** Retrospective two‐dimensional verification of patient treatment plans using multiple CT calibration curves with different gamma analysis criteria.

Patient ID	Anatomical site	Calibration curve	Gamma (3 mm/3%)	Gamma (2 mm/3%)	Gamma (2 mm/2%)
1	Thoracic (low density/lung tissue)	DirectDensity	94.8	90.7	80.2
		120 kV	97.6	95.6	89.5
		Hybrid	98.0	96.4	89.5
2	Abdominal soft tissue (moderate density/spleen)	DirectDensity	93.9	91.3	77.3
		120 kV	97.4	96.5	88.6
		Hybrid	98.3	97.4	90.4
3	Pelvic bone (high density/cortical tissue)	DirectDensity	97.3	96.3	79.3
		120 kV	98.4	98.4	87.2
		Hybrid	98.9	98.9	92.0
4	Thoracic bone (high density/low density)	DirectDensity	96.4	94.8	71.2
		120 kV	96.8	96.4	74.0
		Hybrid	97.6	96.8	82.4

Abbreviations: ID, identification; kV, kilovoltage.

## 3 DESCRIPTION OF THE ERROR AND ITS IMPACT

In the original version of this article, a minor labeling inconsistency was identified in Table 2 and one corresponding sentence in the Results section. The gamma criterion was inadvertently stated as 3 mm/2% instead of 2 mm/3%. The Methods section correctly defines the applied criterion (2 mm/3%, in line with TG‐218), and all figures and associated text, as well as the numerical values reported in the table, correspond to this correct analysis. This does not affect any results, analyses, or conclusions presented in the manuscript. Table 2 and the corresponding text have been corrected accordingly.

We apologize for this error.

